# The association between PM_2.5_ and frailty: evidence from 122 cities in China and 7 countries in Europe

**DOI:** 10.1186/s12889-024-21121-4

**Published:** 2024-12-30

**Authors:** Yanchao Wen, Guiming Zhu, Kexin Cao, Jie Liang, Xiangfeng Lu, Tong Wang

**Affiliations:** 1 Department of Health Statistics, School of Public Health, Shanxi Medical University, 56 XinJian South Road Street, Taiyuan, Shanxi China; 2 Key Laboratory of Coal Environmental Pathogenicity and Prevention (Shanxi Medical University), Ministry of Education, Taiyuan, Shanxi China; 3 Key Laboratory of Cardiovascular Epidemiology, Department of Epidemiology, Fuwai Hospital, Chinese Academy of Medical Sciences and Peking Union Medical College/National Center for Cardiovascular Diseases, Beijing, China

**Keywords:** PM_2.5_, Frailty index, Air pollution, Aging, Cohort study

## Abstract

**Background:**

The accelerated aging process worldwide is placing a heavy burden on countries. PM_2.5_ particulate matter exposure is a significant factor affecting human health and is crucial in the aging process.

**Methods:**

We utilized data from China Health and Retirement Longitudinal Study (CHARLS) and the Survey of Health, Aging, and Retirement in Europe (SHARE) to study the relationship between PM_2.5_ exposure and the frailty index. Acquire PM_2.5_ exposure data for China and Europe, match them according to geographic location within the database. Our study used frailty index to evaluate frailty, which comprises 29 items. We examined the association between PM_2.5_ and frailty index using fixed-effects regression models and Mendelian randomization (MR) analysis.

**Results:**

We first examined the association between PM_2.5_ and frailty index using fixed-effects regression models, revealing a notable positive link across populations in China (coefficient = 0.0003, *P* = 0.0380) and Europe (Coefficient = 0.0019, *P* < 0.0001). This suggests that PM_2.5_ exposure is a significant risk factor for frailty, leading to accelerated frailty. Moreover, our MR analysis uncovered a possible causal association (OR = 1.2933, 95%CI: 1.2045–1.3820, *P* < 0.0001) between PM_2.5_ exposure and the frailty index.

**Conclusions:**

Our findings indicate that long-term exposure to PM_2.5_ in the environment is a risk factor for physical frailty and may have a potential causal relationship. Given the rapid global aging trend, public health measures are needed to reduce PM_2.5_ concentrations and prevent frailty.

**Supplementary Information:**

The online version contains supplementary material available at 10.1186/s12889-024-21121-4.

## Introduction

Aging is evolving into a worldwide trend, this is the development challenge faced by countries worldwide. The World Health Organization (WHO) projects that the elderly population will increase to 1.4 billion by 2030 and 2.1 billion by 2050 [1]. As a result, the rapid growth of the frail elderly population will exert significant pressure on global medical infrastructure and the elderly themselves [2]. Frailty, characterized by a weakened ability to withstand health stress, often impacts the elderly and is linked to numerous negative health outcomes, including all-cause mortality [3], newly diagnosed chronic diseases [4], depressive symptoms [5], and falls [6]. Additionally, research indicates that active improvement strategies can mitigate frailty [7, 8], improving the quality of life for the elderly.

Air pollution, as the greatest environmental risks to health, may contribute to the aging process and impact successful aging, which is especially significant in understanding aging [9]. In 2019, a staggering 99% of people worldwide resided in areas failing to comply with WHO’s air quality criteria [10]. Air pollution’s health impacts are diverse, encompassing respiratory diseases, allergic conditions [11], cardiovascular disorders [12], and brain health [13]. Epidemiological evidence indicates a potential link between these diseases and both long-term exposure to air pollution and frailty [14–17].

In recent years, several studies have investigated the impact of air pollution on frailty. For example, research has reported a 30% increase in the likelihood of frailty for every 10 µg/m^3^ rise in PM_2.5_ levels in rural areas [18]. A population-based quasi-experimental study, utilizing propensity score matching and double difference analysis, showed a significant decrease in individuals’ FI scores and the frailty status improved following the implementation of the Clean Air Action in China [19]. However, current research, despite their contributions, exhibits several limitations. Firstly, the evidence predominantly stems from cross-sectional studies, which inherently lack the capacity to establish causal relationships. However, research has shown that cohort studies and Mendelian randomization (MR) studies provide more convincing evidence than cross-sectional studies [20]. Secondly, the research subjects are primarily concentrated within a single region, resulting in a scarcity of cross-regional studies. Consequently, there is a pressing need for cross-regional research to yield more universally applicable findings. Finally, there is a paucity of research examining the relationship between PM_2.5_ exposure and the Frailty Index (FI). Studies have shown that the FI is widely recognized as a standard measure of aging and can be used to assess the burden of age-related clinically significant health deficits [4, 21]. Furthermore, the FI demonstrates strong predictive ability and is effective in forecasting adverse health outcomes [22].

To investigate the association and causal relationship between PM_2.5_ exposure and FI, we utilized data from the China Health and Retirement Longitudinal Study (CHARLS), the Survey of Health, Ageing and Retirement in Europe (SHARE), and summary data from Genome-Wide Association Studies (GWAS). Employing fixed effect regression models and MR methods, we aimed to provide a comprehensive analysis of the potential impacts of PM_2.5_ on FI.

## Methods

### Study design

**Fig. 1:**
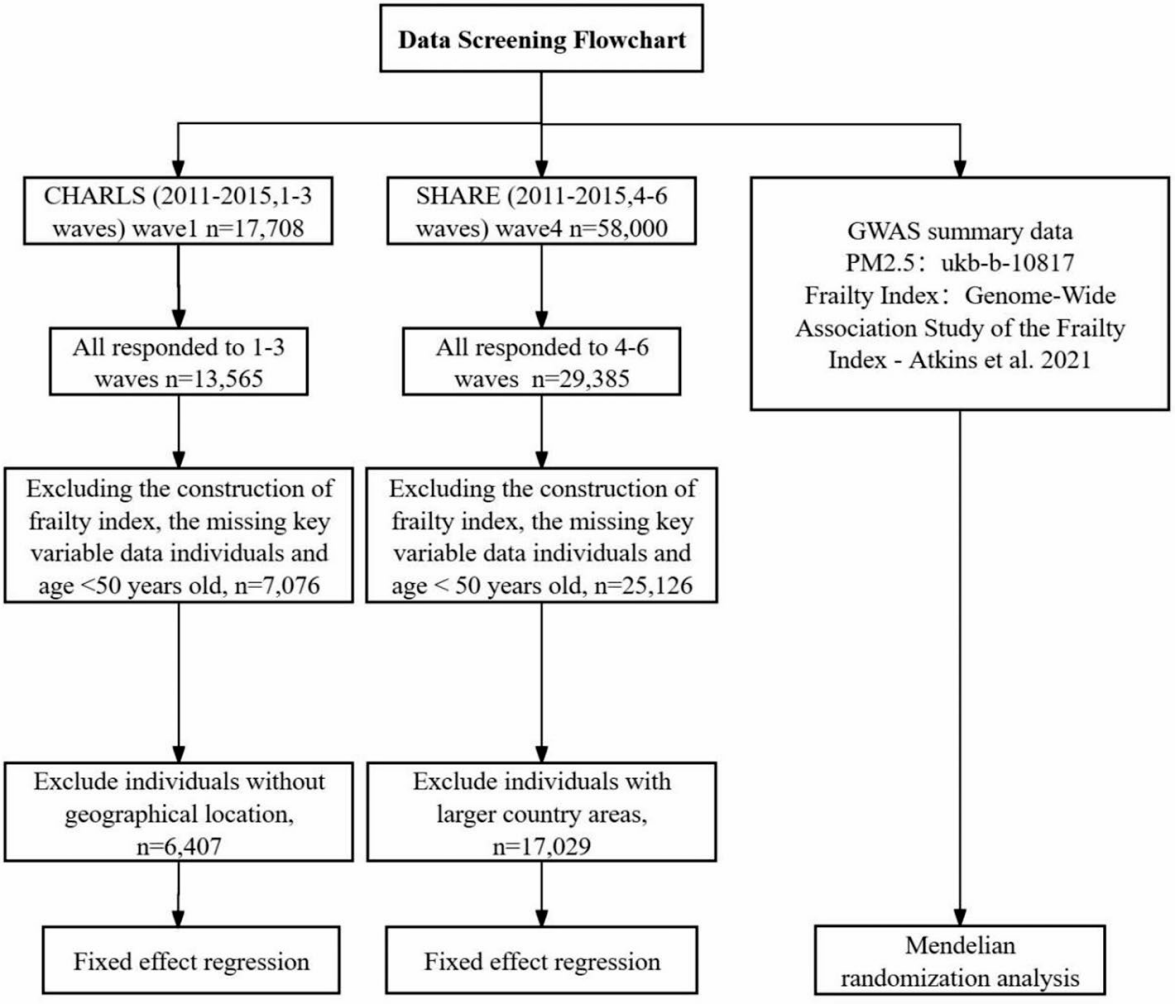
Data screening flowchart

The baseline survey of CHARLS, a nationally representative cohort study of Chinese residents aged 45 and above, began in 2011. It included 17,708 individuals and was followed up every two years [23]. The survey used multistage probability sampling to select respondents. In addition, data from SHARE, the largest longitudinal study in Europe, are available for people aged 50 years and older. We used data from three follow-up visits from 2011 to 2015. We obtained the following information from the two cohorts: gender, age, marital status, education, residence, smoking status, drinking status, retirement status, body mass index (BMI (kg/m^2^)), and a variety of chronic diseases. In addition, information was obtained on variables related to activities of daily living (ADL), instrumental activities of daily living (IADL), physical function limitations, cognitive abilities, and depression. Figure 1 shows the data screening flowchart.

**Fig. 2:**
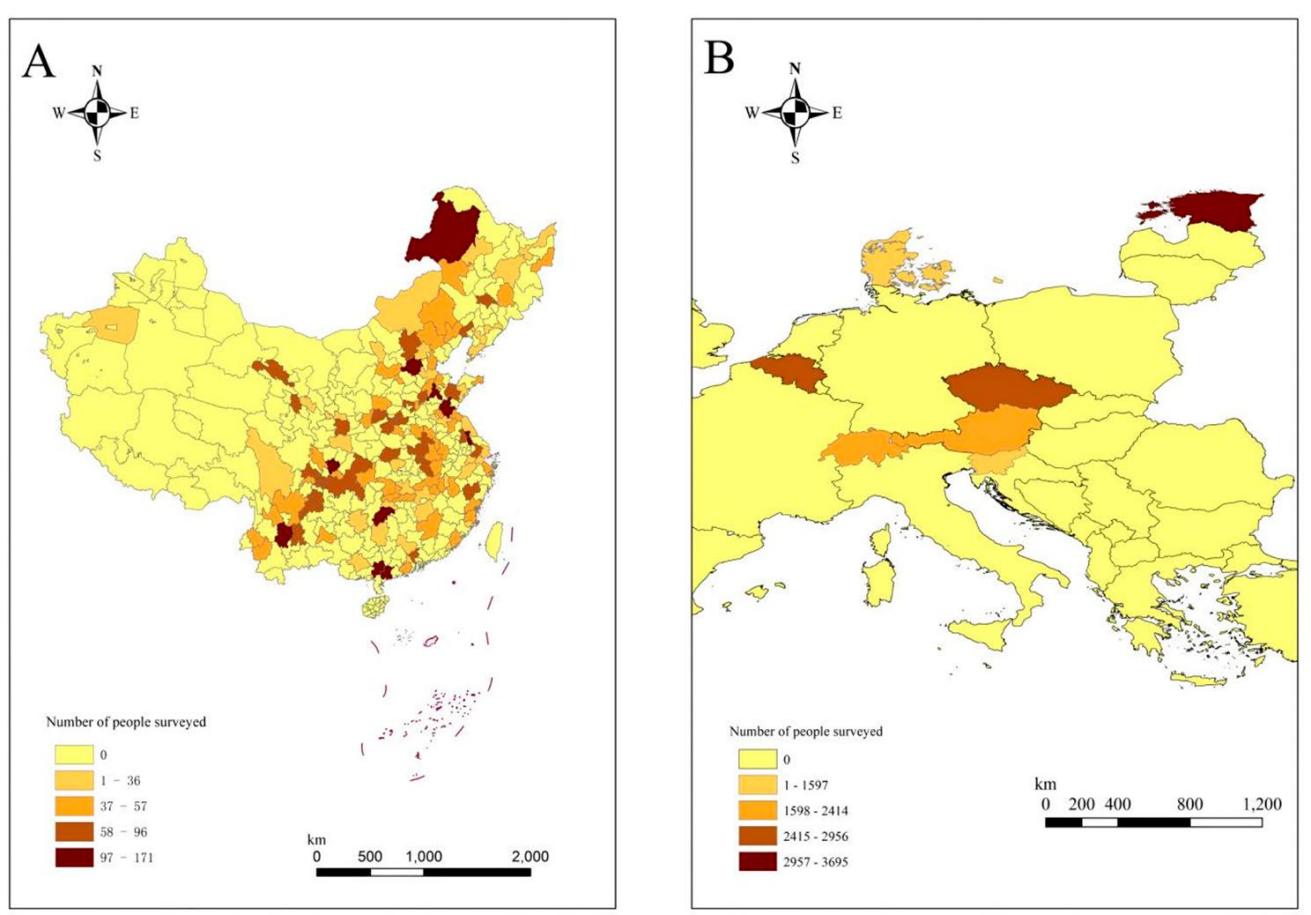
122 Chinese cities and 7 European countries included and the number of respondents. The darker the color, the more respondents were included. **A**: China; **B**: Europe

To ensure study consistency, we limited the age range of both cohorts to those over 50 years of age. During the research process, for CHARLS, we excluded participants who lacked information on key variables for constructing the FI, as well as participants data lacked geographical location information. Finally, we included 6,407 research subjects. The SHARE data is consistent with the CHARLS data above, and participants from European countries with land areas smaller than or comparable to the largest cities in China were retained (include Estonia, Belgium, Czech Republic, Austria, Switzerland, Denmark, Slovenia). Finally, 17,029 research subjects were included. Figure 2 shows the 122 cities in China and 7 countries in Europe included and the number of people surveyed. The darker the color, the more surveyed people are included.

Furthermore, MR analysis was performed using GWAS summary data to explore the causal association between FI and PM_2.5_. The summary data for PM_2.5_ was sourced from the IEU Open GWAS project (ID: ukb-b-10817). For the FI, GWAS data were derived from a study that investigated British individuals of European descent [24, 25]. This study encompassed a GWAS meta-analysis of the FI conducted on Biobank participants and Swedish twin genome participants. Calculations for FI utilized self-reported health-related information from the UK Biobank (UKB) and Twin Genome, encompassing 49 to 44 elements related to symptoms, disability, and identified illnesses.

## Air pollution data

Ambient PM_2.5_ data were obtained from previously established dataset through satellite-based remote sensing technology [26, 27]. The time span of air pollution data is consistent with that of the study population, which is 2011–2015. Specifically, air pollution data for China were sourced from the China High-Resolution, High-Quality Near Surface Air Pollutant dataset published by Wei et al. [26]. This dataset employs a comprehensive approach, integrating big data from various sources such as ground-based measurements, satellite remote sensing products, atmospheric reanalysis, and model simulations. By incorporating artificial intelligence, the dataset effectively generates PM_2.5_ data while accounting for the spatiotemporal heterogeneity of air pollution. Notably, the dataset boasts a spatial resolution of 1 km × 1 km, offering enhanced accuracy and predictive capability compared to previously reported datasets. To integrate the mean concentration of each grid cell with geographic data, ArcGIS software (ESRI Inc.) was utilized. This process involved using a geographic shapefile that contained the boundaries of prefecture-level cities in mainland China. Therefore, we were able to match and calculate the average PM_2.5_ concentration levels across 122 cities. These calculations were based on data collected from three follow-up visits conducted between 2011 and 2015.

**Fig. 3:**
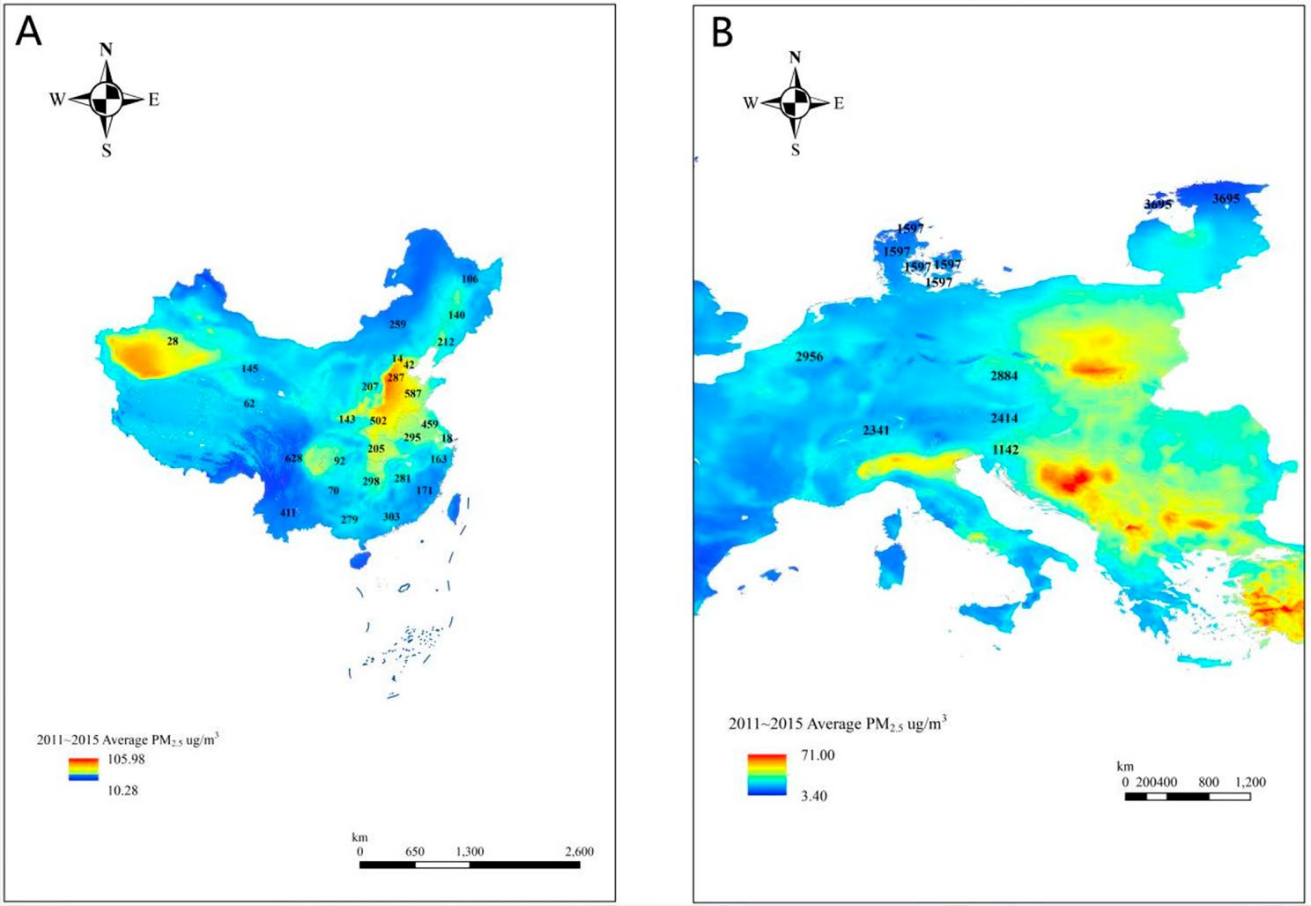
The average PM_2.5_ concentrations from 2011 to 2015 across 122 cities in China and 7 European countries. The numbers represent the number of people surveyed included from each provincial-level unit in China or each European country. **A**: China; B: Europe

European PM_2.5_ concentrations were obtained from the Atmospheric Composition Analysis Group Web site at Washington University, which is a 1 km × 1 km global model developed by HAMMER et al. [27]. HAMMER and colleagues combined aerosol optical depth (AOD) retrievals from NASA’s MODIS, MISR, SeaWIFS, and VIIRS with the GEOS-Chem chemical transport model. They then used a residual convolutional neural network (CNN) to calibrate these results against global ground observations, estimating annual and monthly surface PM_2.5_ from 2000 to 2019. Similar to China, to facilitate the matching of PM_2.5_ data for seven European countries, country-level geographic location information was obtained from SHARE. Figure 3 shows the average PM_2.5_ concentrations from 2011 to 2015 across 122 cities in China and 7 European countries. The numbers represent the number of people surveyed included from each provincial-level unit in China or each European country.

In addition to PM_2.5_ concentration, NO_2_ concentration and temperature levels are considered influential factors. Therefore, global NO_2_ concentrations (with a spatial resolution of 1 km × 1 km) [28] and temperature data (https://cds.climate.copernicus.eu/cdsapp#!/dataset/reanalysis-era5-pressure-levels) were incorporated as control variables in the model. This comprehensive approach ensures a thorough examination of the impact of PM_2.5_ concentration while accounting for potential confounding effects of NO_2_ concentration and temperature levels.

## Assessment of the frailty index

In our study, we used the FI to evaluate frailty, which is characterized by the accumulation of various age-related health problems. During the follow-up period between 2011 and 2015, three repeated measurements of FI were taken for individual. The FI was developed according to established procedures and principles outlined by Searle SD [29, 30], and informed by relevant previous research [3, 31]. Based on data from CHARLS and SHARE, a total of 29 items were selected for the construction of the FI. These items included illness, disability in ADL and disability in IADL, physical functioning, depression, and cognition. Each item was scored as 1 (deficit present) or 0 (no deficit), respectively. The scores for items 1–29 were summed to obtain the FI score, which ranged from 0 to 29. Items 28 and 29 representing cognitive and depression scores, are continuous variables, their values spanning from 0 to 1. Currently, research focuses on categorizing FI. However, existing studies have revealed discrepancies in the classification of FI and in identifying older adults as “frail”, thereby limiting our understanding of frailty. Maintaining FI as a continuous variable may be beneficial until further research establishes the optimal FI category for this population [32]. Therefore, the FI for each participant is calculated by dividing the total current health deficits by 29. As a continuous variable, FI ranges from 0 to 1, with higher values indicating greater frailty. The variables for constructing the FI are shown in the Supplementary table (Supplementary Table 1).

### Statistical analysis

After obtaining air pollution exposure data and FI outcome data, we matched them based on the geographic location information provided by the CHARLS and SHARE databases. The data for each individual included three PM_2.5_ concentration data and FI measurements, as well as a series of covariate data. First, to reduce the impact of missing values on the analysis, we excluded individuals with missing information on key variables in the construction of the FI. For the remaining individuals, we performed multiple imputation of covariates to obtain complete data for analysis [33, 34]. The proportion of missing variables is shown in the supplementary table (Supplementary Table 2). Fixed effects regression was used to evaluate the longitudinal data. Fixed effects regression can account for both observed and unobserved time-invariant confounding variables [35]. Consequently, fixed effect regression is deemed more robust than conventional regression models in investigating the correlation between predictor alterations and outcome variations.

The fixed effect regression model is as follows

$${{\rm{Y}}_{it}}{\rm{ = }}\alpha {\rm{ + }}{\alpha _i}{\rm{ + }}{\beta _1}{X_{1it}} + {\beta _2}{X_{2it}} +...{\beta _k}{X_{kit}} + {\varepsilon _{it}}$$

$\:{\varvec{Y}}_{\varvec{i}\varvec{t}}\:$is the dependent variable (the FI in this study), $\:{\varvec{X}}_{\varvec{k}\varvec{i}\varvec{t}}\:$is the independent variable and the covariate that needs to be controlled (such as PM_2.5_ and BMI in this study), $\:\varvec{\alpha\:}\:$is the intercept term,$\:\:{\varvec{\alpha\:}}_{\varvec{i}\:}$is the individual fixed effect, $\:{\varvec{\epsilon}}_{\varvec{i}\varvec{t}}\:$is the error bar, and $\:{\varvec{\beta\:}}_{\varvec{k}}$ is the regression coefficient.

European SHARE data was employed to explore the impact of regional variances on the link between PM_2.5_ and FI. The following covariates were controlled for in the analysis: age, marital status, education, smoking status, drinking status, retirement status, BMI, mean annual NO_2_ concentration and temperature.

Furthermore, to explore the causal association between the two variables, we performed MR analysis using GWAS summary data. In this analysis, we initially filtered out outlier single nucleotide polymorphisms (SNPs), retaining only those SNPs deemed reliable for further investigation. The MR analysis was primarily conducted employing the inverse variance weighted with modified weights (MW-IVW) method [36] as the main analytical approach. Additionally, sensitivity analyses were performed using the Inverse Variance Weighted (IVW) [37] and weighted median (WM) methods [38] to assess the robustness of the findings. To test for pleiotropy, the MR-Egger method was employed, and the F statistic was applied to gauge the strength of instrumental variables in the study [39, 40].

The statistical evaluations were performed utilizing R (version 4.2.3), ArcGIS (version 10.8), Stata 16, and SPSS 25 software, considering a *P*-value less than 0.05 as statistically significant.

## Results

### Basic characteristics of study participants

The baseline basic characteristics of CHARLS and SHARE participants are presented in Table 1. The mean age (SD) of 6,407 participants in CHARLS was 60.67 years (7.11 years), with males comprising 49.9%. And the proportion of elderly people aged 65 and above is 25.63%. The mean annual exposure concentration of PM_2.5_ in 2011 at baseline was 58.1757 µg/m^3^, the average FI in 2011 was 0.1268. The mean age (SD) of all 17,029 participants in SHARE was 65.06 years (9.13 years), with males comprising 41.2% and over 65 years old 46.63%. The mean annual exposure concentration of 14.8600 µg/m^3^ of PM_2.5_ in 2011 at baseline, the average FI in 2011 was 0.1037.

**Table 1:** Fundamental traits of the population studied

Characteristic	CHARLS (*N* (%))	SHARE (*N* (%))
Total sample (N)	6,407	17,029
Males	3,194 (49.9%)	6,992 (41.1%)
≥ 65 years old	1,706 (26.63%)	7,940 (46.63%)
Age (Mean ± SD, years)	60.67 ± 7.11	65.06 ± 9.13
BMI (Mean ± SD, years)	23.42 ± 3.84	27.11 ± 4.80
Marital status (married)	5,964 (88.9%)	11,062 (65.0%)
Educational level		
Less than lower secondary	5,740 (89.9%)	5,003 (29.4%)
Upper secondary & vocational training	594 (9.3%)	7,785 (45.7%)
Tertiary	72 (1.1%)	4,341 (24.9%)
Residence (Rural)	4,178 (65.2%)	5,993 (35.2%)
Smoking status (Smoker)	2,009 (32.8%)	3,348 (19.7%)
Drinking status (Drinker)	2,191 (34.2%)	8,081 (47.1%)
Frailty Index in 2011 (Mean ± SD)	0.1268 ± 0.1078	0.1037 ± 0.1031
PM_2.5_ in 2011 (Mean ± SD, µg/m^3^)	58.1757 ± 17.8567	14.8600 ± 3.6226
NO_2_ in 2011 (Mean ± SD, µg/m^3^)	7.1756 ± 4.5943	5.3475 ± 2.3394
Temperature in 2011 (Mean ± SD, ℃)	13.9071 ± 5.0140	8.2235 ± 1.6146

## Association between PM_2.5_ and Frailty Index in cohort study

In Table 2, the association between PM_2.5_ and the FI is displayed. Initially, a significant positive correlation was identified without controlling for potential confounding variables: The FI increases by 0.0030 for every 10 µg/m^3^ rise in PM_2.5_ concentration, *P* = 0.0290. Upon adjusting for these confounding factors, a positive relationship between PM_2.5_ and the FI persisted (coefficient = 0.0003, *P* = 0.0380). This suggests that with each 10 µg/m^3^ rise in PM_2.5_ concentration in the atmosphere, the FI also increases by 0.0030. In the SHARE data, we observed similar results. Prior to adjustment: coefficient = 0.0022, *P* < 0.0001, after adjustment: coefficient = 0.0019, *P* < 0.0001. This indicates that the FI was found to increase by 0.0190 for every 10 µg/m^3^ increase in PM_2.5_ concentration. Limiting the study to individuals aged 65 years and older revealed that PM_2.5_ remained a risk factor for frailty in the European population. While a positive association was observed in the Chinese population, it was not statistically significant (*P* = 0.3140). Additionally, to further explore the urban-rural associations. CHARLS data revealed a notable positive relationship between PM_2.5_ and the FI in rural areas: coefficient = 0.0004, *P* = 0.0250. However, no such association was found in urban areas: coefficient = 0.0001, *P* = 0.6220. In the SHARE, the impact of PM_2.5_ on the FI remains consistent, indicating that it serves as a risk factor for frailty.

## Casual association between PM_2.5_ and Frailty Index in MR

The F-statistic (MeanF = 23.9521) and MR-Egger results (*P* = 0.5290) demonstrated that the analysis was not influenced by weak instrumental variables and pleiotropy. The MR results indicated that MWIVW: Odds ratio (OR) = 1.2933, 95% CI: 1.2045–1.3820, *P* < 0.0001; the results indicated that PM_2.5_ was a significant risk for the FI. Sensitivity analyses showed similar results: IVW: OR = 1.2820, 95% CI: 1.1874–1.3767, *P* < 0.0001; WM: OR = 1.2630, 95% CI: 1.1271–1.3989, *P* = 0.0008. Our findings demonstrate that higher PM_2.5_ levels expedite the frailty process., with each standard deviation increase in PM_2.5_ associated with a 29.33% rise in frailty risk.

**Table 2:** Main results

Method	CHARLS		SHARE	
	Coefficient	*P*	Coefficient	*P*
Fixed effects regression (Model 1)	0.0003	0.0290	0.0022	< 0.0001
Fixed effects regression (Model 2)	0.0003	0.0380	0.0019	< 0.0001
Fixed effects regression (Rural)	0.0004	0.0250	0.0031	< 0.0001
Fixed effects regression (Urban)	0.0001	0.6220	0.0016	0.0005
Fixed effects regression (≥ 65 years old)	0.0003	0.3140	0.0025	< 0.0001
MR	$\:\text{O}\text{R}\:$= 1.2933, 95%CI: 1.2045–1.3820, *P* < 0.0001			

Note: Model 1 is not adjusted. Model 2 adjusted for age, marital status, education, smoking status, drinking status, retirement status, BMI, mean annual NO_2_ concentration and temperature. OR: Odds ratio; CHARLS: the China Health and Retirement Longitudinal Study; SHARE: the Survey of Health, Aging and Retirement in Europe; MR: Mendelian randomization

## Discussion

The longitudinal study demonstrated a significant positive association between PM_2.5_ exposure and FI, and MR results indicated a causal association. Our study investigated the association between PM_2.5_ and FI through a follow-up design. It is noteworthy that a generally accepted instrument was used to measure frailty, and the FI was measured using a combination of 29 items. Through cross-ethnic studies, we obtained similar results in European populations, indicating that exposure to PM_2.5_ can accelerate frailty in people of different ethnic groups. Our study revealed that exposure to PM_2.5_ may speed up the aging process, similar findings were observed across different regions, and suggesting a possible causal relationship.

Numerous research efforts have focused on exploring the link between air pollution and frailty among older adults. Research involving prospective cohorts revealed a correlation between air pollutants, like PM_2.5_, and a heightened likelihood of frailty [41]. Consistent with the finding, a population-based study involving 220,079 UKB participants revealed that higher exposure to PM_2.5_ was associated with an elevated risk of frailty [42]. Likewise, studies derived from the Chinese Longitudinal Healthy Longevity Survey revealed an increased occurrence of frailty linked to heightened exposure to air pollution in the year preceding the interview [43]. Notably, frailty scores were significantly higher in older adults residing in areas with severe air pollution. This implies that air contamination could be a major factor in shaping the progression of healthy aging [43]. Furthermore, after China implemented the air pollution control policy, namely the Clean Air Action Plan, the FI scores of healthy individuals were significantly reduced by 0.0205, while the FI scores of pre-frail individuals were significantly reduced by 0.0114 [19]. Although these studies support the adverse effects of ambient air pollution on frailty, they do not include cross-regional research or explore causal relationships. Building on a long-term cohort study, we incorporated a MR study, resulting in findings that are more robust compared to existing results. Our research corroborates the negative impact of environmental air pollution on frailty. Exposure to PM_2.5_ is positively correlated with the FI, thereby accelerating the aging process. Interestingly, when we restricted our study subjects to individuals aged 65 and above, we observed a positive correlation between PM_2.5_ exposure and FI. However, in the sample of the elderly population in China, this association did not reach statistical significance. This phenomenon may be related to significant lifestyle differences between elderly populations in China and Europe, which could influence the level of PM_2.5_ exposure among those aged 65 and above and differences in genetic backgrounds and physiological characteristics might also contribute to this phenomenon [44–46]. In addition, our investigation brought to light disparities in results between China rural and urban settings. One potential explanation for this variation is that rural regions heavily rely on traditional energy sources like biomass burning, leading to higher levels of both outdoor and indoor air pollution [18]. Moreover, compared to urban residents, rural areas lack proper housing and transportation planning, which may exacerbate environmental exposures for rural residents and result in their limited understanding of the significance of air pollution prevention and control [47, 48].

Exposure to air pollution is widely recognized for causing a range of detrimental health impacts, including inflammatory reactions, oxidative stress, metabolic disorders, and epigenetic modifications. For instance, by upsetting mitochondria, air pollution can cause pro-inflammatory reactions in different immune cells, and since inflammation is thought to be a possible source of weakness, thus collectively leading to the onset of weakness [49, 50]. Moreover, air pollutants may disrupt the body’s balance and reduce its ability to handle stress, hastening the decline in functional abilities and capacities associated with aging levels of cells, organs, and the entire system, ultimately resulting in frailty [7]. Clearly, air pollution plays a role in frailty to a certain extent, making the reduction of air pollution crucial for diminishing frailty in the elderly. With the rapid development of the global economy, environmental pollution and population aging have emerged as two critical issues impacting public health. Air pollution, a pervasive and increasingly severe environmental hazard, has long been a significant factor contributing to chronic diseases such as respiratory disorders, cardiovascular diseases, and cancer [51]. This issue is particularly pronounced in regions experiencing rapid industrialization, where declining air quality poses a substantial threat to public health. Concurrently, the global population is aging at an accelerated pace due to advancements in healthcare and declining birth rates, leading to an increasing proportion of elderly individuals [52]. This demographic shift not only alters the population structure but also presents challenges in healthcare, social security, and lifestyle adjustments. The elderly population typically faces a higher burden of chronic diseases, and as they age, their immune and recovery capacities diminish, heightening their sensitivity to environmental pollution [53]. The interplay between air pollution and aging exacerbates public health pressures. Therefore, based on current research findings, it is imperative to implement measures to mitigate the impact of air pollution on the aging population. Such measures could include the development of effective public health policies by governments, enhancement of urban green spaces [54], dissemination of knowledge regarding air pollution and health risks to the public, and provision of personalized healthcare services for the elderly [55].

The present study has several significant strengths. Firstly, a cohort study design was employed to investigate the longitudinal association between PM_2.5_ and FI in depth. During the research process, the effects of factors such as temperature, NO_2_ and important covariates were carefully controlled to ensure the reliability of the results. Secondly, we constructed a comprehensive FI, taking into account multiple factors, including disease, physical functional limitations, disability in ADL, disability in IADL, physical function, depression, and cognition, to comprehensively assess the FI. Finally, we utilized data from CHARLS and SHARE. Moreover, we ensured consistency in variables used to construct the FI between SHARE and CHARLS, with a consistent data timeframe from 2011 to 2015. Through cross-regional observations, we obtained consistent results. Additionally, we further established causal relationship through MR analysis. Therefore, our study findings are generalizable and demonstrate the impact of PM_2.5_ on frailty, providing robust support for the credibility of our research.

Although our study yielded some important findings, its limitations must also be acknowledged. First, the pollutant data used in the study are based on city-level data, which may not fully capture small changes within cities. The lack of consideration for variability within the city may introduce bias into the results, as detailed geographic locations could more accurately capture the study subjects’ exposure levels, leading to more precise research outcomes. Second, we used a validated tool to detect FI, but we adapted it based on information available in the research database used. The use of existing data may introduce bias from the original version. Third, to ensure the maximum inclusion of the sample size, we performed multiple imputation of cognitive variables required to construct FI in the SHARE database (such as: Orient variable: missing proportion: wave 4: 28.32%, wave 5: 99.62%, wave 6: 0.23%), which may have some impact on the results. However, the multiple imputation method we employed is widely recognized for addressing missing data in cohort studies, and its effectiveness is well-documented [34, 56]. Finally, we acknowledge that the data from CHARLS and SHARE may not fully represent populations in other regions with different socioeconomic or environmental context. However, our research indicates that exposure to PM_2.5_ accelerates aging in both China and Europe, suggesting that the adverse health effects of PM_2.5_ are widespread to some extent. Therefore, it is important to be cautious in interpreting the findings and to address these limitations in future studies.

## Conclusion

In conclusion, our research indicates that prolonged exposure to PM_2.5_ is a risk factor for frailty and has a potential causal relationship. Therefore, in the context of an aging population, it is crucial to address the adverse health effects of air pollution. Effective public health measures should be implemented to reduce the concentration of environmental particulates, such as PM_2.5_, while also mitigating the aging process to enhance overall public health.

## Electronic supplementary material

Below is the link to the electronic supplementary material.

Supplementary Material 1

## Data Availability

The China Health and Retirement Longitudinal Study (CHARLS): https://charls.charlsdata.com/pages/data/111/en.html. The Survey of Health, Ageing and Retirement in Europe (SHARE): http://www.share-project.org/data-access.html. Harmonized data for CHARLS and SHARE can be accessed via: https://g2aging.org/hrd/get-data. GWAS summary data for PM_2.5_: https://gwas.mrcieu.ac.uk/datasets/ukb-b-10817/. GWAS summary data for frailty index:https://figshare.com/articles/dataset/Genome-Wide_Association_Study_of_the_Frailty_Index_-_Atkins_et_al_2019/9204998

## References

[1] http://www.who.int/news-room/fact-sheets/detail/ageing-and-health2022; Aging and Health.

[2] Dent E, Martin FC, Bergman H, Woo J, Romero-Ortuno R, Walston JD. Management of frailty: opportunities, challenges, and future directions. Lancet. 2019;394(10206):1376–86. 31609229 10.1016/S0140-6736(19)31785-4

[3] Fan J, Yu C, Guo Y, Bian Z, Sun Z, Yang L, Chen Y, Du H, Li Z, Lei Y, et al. Frailty index and all-cause and cause-specific mortality in Chinese adults: a prospective cohort study. Lancet Public Health. 2020;5(12):e650–60. 33271078 10.1016/S2468-2667(20)30113-4 PMC7708389

[4] Jang J, Jung H, Shin J, Kim DH. Assessment of Frailty Index at 66 years of Age and Association with Age-Related diseases, disability, and Death over 10 years in Korea. JAMA Netw Open. 2023;6(3):e2248995. 36862415 10.1001/jamanetworkopen.2022.48995 PMC9982694

[5] Zhu J, Zhou D, Nie Y, Wang J, Yang Y, Chen D, Yu M, Li Y. Assessment of the bidirectional causal association between frailty and depression: a mendelian randomization study. J Cachexia Sarcopenia Muscle. 2023;14(5):2327–34. 37670569 10.1002/jcsm.13319 PMC10570069

[6] de Vries OJ, Peeters GM, Lips P, Deeg DJ. Does frailty predict increased risk of falls and fractures? A prospective population-based study. Osteoporos Int. 2013;24(9):2397–403. 23430104 10.1007/s00198-013-2303-z

[7] Clegg A, Young J, Iliffe S, Rikkert MO, Rockwood K. Frailty in elderly people. Lancet. 2013;381(9868):752–62. 23395245 10.1016/S0140-6736(12)62167-9 PMC4098658

[8] Dent E, Kowal P, Hoogendijk EO. Frailty measurement in research and clinical practice: a review. Eur J Intern Med. 2016;31:3–10. 27039014 10.1016/j.ejim.2016.03.007

[9] Cohen G, Gerber Y. Air Pollution and successful aging: recent evidence and New perspectives. Curr Environ Health Rep. 2017;4(1):1–11. 28101729 10.1007/s40572-017-0127-2

[10] https://www.who.int/news-room/fact-sheets/detail/ambient-(outdoor)-air-quality-and-health, Ambient (outdoor) air pollution.

[11] Sierra-Vargas MP, Teran LM. Air pollution: impact and prevention. Respirology. 2012;17(7):1031–8. 22726103 10.1111/j.1440-1843.2012.02213.x PMC3532603

[12] Lee KK, Miller MR, Shah ASV. Air Pollution and Stroke. J Stroke. 2018;20(1):2–11. 29402072 10.5853/jos.2017.02894 PMC5836577

[13] Russ TC, Reis S, van Tongeren M. Air pollution and brain health: defining the research agenda. Curr Opin Psychiatry. 2019;32(2):97–104. 30543549 10.1097/YCO.0000000000000480

[14] Chen H, Goldberg MS, Villeneuve PJ. A systematic review of the relation between long-term exposure to ambient air pollution and chronic diseases. Rev Environ Health. 2008;23(4):243–97. 19235364 10.1515/reveh.2008.23.4.243

[15] Peters R, Peters J, Booth A, Mudway I. Is air pollution associated with increased risk of cognitive decline? A systematic review. Age Ageing. 2015;44(5):755–60. 26188335 10.1093/ageing/afv087

[16] Vetrano DL, Palmer K, Marengoni A, Marzetti E, Lattanzio F, Roller-Wirnsberger R, Lopez Samaniego L, Rodríguez-Mañas L, Bernabei R, Onder G. Frailty and Multimorbidity: a systematic review and Meta-analysis. J Gerontol Biol Sci Med Sci. 2019;74(5):659–66. 10.1093/gerona/gly110 29726918

[17] Pilotto A, Custodero C, Maggi S, Polidori MC, Veronese N, Ferrucci L. A multidimensional approach to frailty in older people. Ageing Res Rev. 2020;60:101047. 32171786 10.1016/j.arr.2020.101047 PMC7461697

[18] Guo YF, Ng N, Kowal P, Lin H, Ruan Y, Shi Y, Wu F. Frailty Risk in older adults Associated with Long-Term exposure to ambient PM2.5 in 6 Middle-Income Countries. J Gerontol Biol Sci Med Sci. 2022;77(5):970–6. 10.1093/gerona/glac022 PMC9071498 35134914

[19] Guo Y, Yang F. Effects of China’s Clean Air Act on Frailty Levels Among Middle-Aged and Older Adults: A Population-Based Quasi-Experimental Study. J Gerontol Biol Sci Med Sci 2024;79(4). 10.1093/gerona/glae040 38330396

[20] Arsenault BJ. From the garden to the clinic: how mendelian randomization is shaping up atherosclerotic cardiovascular disease prevention strategies. Eur Heart J. 2022;43(42):4447–9. 35869924 10.1093/eurheartj/ehac394

[21] Mitnitski AB, Mogilner AJ, Rockwood K. Accumulation of deficits as a proxy measure of aging. ScientificWorldJournal. 2001;1:323–36. 12806071 10.1100/tsw.2001.58 PMC6084020

[22] Kaskirbayeva D, West R, Jaafari H, King N, Howdon D, Shuweihdi F, Clegg A, Nikolova S. Progression of frailty as measured by a cumulative deficit index: a systematic review. Ageing Res Rev. 2023;84:101789. 36396032 10.1016/j.arr.2022.101789

[23] Zhao Y, Hu Y, Smith JP, Strauss J, Yang G. Cohort profile: the China Health and Retirement Longitudinal Study (CHARLS). Int J Epidemiol. 2014;43(1):61–8. 23243115 10.1093/ije/dys203 PMC3937970

[24] Pilling L et al. Genome-Wide Association Study of the Frailty Index - Atkins. 2021. figshare. Dataset. 2019. 10.1111/acel.13459 PMC8441299 34431594

[25] Atkins JL, Jylhävä J, Pedersen NL, Magnusson PK, Lu Y, Wang Y, Hägg S, Melzer D, Williams DM, Pilling LC. A genome-wide association study of the frailty index highlights brain pathways in ageing. Aging Cell. 2021;20(9):e13459. 34431594 10.1111/acel.13459 PMC8441299

[26] Wei Jing LIZ. ChinaHighPM_2.5_: High-resolution and High-quality Ground-level PM_2.5_ Dataset for China (2000–2023). In. Edited by National Tibetan Plateau Data C: National Tibetan Plateau Data Center; 2024.

[27] Hammer MS, van Donkelaar A, Li C, Lyapustin A, Sayer AM, Hsu NC, Levy RC, Garay MJ, Kalashnikova OV, Kahn RA, et al. Global estimates and long-term trends of fine particulate matter concentrations (1998–2018). Environ Sci Technol. 2020;54(13):7879–90. 32491847 10.1021/acs.est.0c01764

[28] Mohegh A, Anenberg S. Global surface NO2 concentrations 1990–2020; figshare. Dataset. In.; 2020.

[29] Searle SD, Mitnitski A, Gahbauer EA, Gill TM, Rockwood K. A standard procedure for creating a frailty index. BMC Geriatr. 2008;8:24. 18826625 10.1186/1471-2318-8-24 PMC2573877

[30] Searle SD, Rockwood K. What proportion of older adults in hospital are frail? Lancet. 2018;391(10132):1751–2. 29706363 10.1016/S0140-6736(18)30907-3

[31] He D, Wang Z, Li J, Yu K, He Y, He X, Liu Y, Li Y, Fu R, Zhou D, et al. Changes in frailty and incident cardiovascular disease in three prospective cohorts. Eur Heart J. 2024;45(12):1058–68. 38241094 10.1093/eurheartj/ehad885

[32] Fletcher JA, Logan B, Reid N, Gordon EH, Ladwa R, Hubbard RE. How frail is frail in oncology studies? A scoping review. BMC Cancer. 2023;23(1):498. 37268891 10.1186/s12885-023-10933-z PMC10236730

[33] Van Buuren S. Flexible Imputation of Missing Data, Second Edition edn: Chapman & Hall/CRC. Boca Raton, FL.; 2018.

[34] Huque MH, Carlin JB, Simpson JA, Lee KJ. A comparison of multiple imputation methods for missing data in longitudinal studies. BMC Med Res Methodol. 2018;18(1):168. 30541455 10.1186/s12874-018-0615-6 PMC6292063

[35] Isong IA, Richmond T, Kawachi I, Avendaño M. Childcare attendance and obesity risk. Pediatrics 2016;138(5). 10.1542/peds.2016-1539 PMC5079080 27940780

[36] Bowden J, Del Greco MF, Minelli C, Zhao Q, Lawlor DA, Sheehan NA, Thompson J, Davey Smith G. Improving the accuracy of two-sample summary-data mendelian randomization: moving beyond the NOME assumption. Int J Epidemiol. 2019;48(3):728–42. 30561657 10.1093/ije/dyy258 PMC6659376

[37] Burgess S, Butterworth A, Thompson SG. Mendelian randomization analysis with multiple genetic variants using summarized data. Genet Epidemiol. 2013;37(7):658–65. 24114802 10.1002/gepi.21758 PMC4377079

[38] Bowden J, Davey Smith G, Haycock PC, Burgess S. Consistent estimation in mendelian randomization with some Invalid instruments using a weighted median estimator. Genet Epidemiol. 2016;40(4):304–14. 27061298 10.1002/gepi.21965 PMC4849733

[39] Pierce BL, Ahsan H, Vanderweele TJ. Power and instrument strength requirements for mendelian randomization studies using multiple genetic variants. Int J Epidemiol. 2011;40(3):740–52. 20813862 10.1093/ije/dyq151 PMC3147064

[40] Bowden J, Davey Smith G, Burgess S. Mendelian randomization with invalid instruments: effect estimation and bias detection through Egger regression. Int J Epidemiol. 2015;44(2):512–25. 26050253 10.1093/ije/dyv080 PMC4469799

[41] Guo X, Su W, Wang X, Hu W, Meng J, Ahmed MA, Qu G, Sun Y. Assessing the effects of air pollution and residential greenness on frailty in older adults: a prospective cohort study from China. Environ Sci Pollut Res Int. 2024;31(6):9091–105. 38183550 10.1007/s11356-023-31741-9

[42] Veronese N, Maniscalco L, Matranga D, Lacca G, Dominguez LJ, Barbagallo M. Association between Pollution and Frailty in Older people: a cross-sectional analysis of the UK Biobank. J Am Med Dir Assoc. 2023;24(4):475–e481473. 36774967 10.1016/j.jamda.2022.12.027

[43] Hu K, Keenan K, Hale JM, Börger T. The association between city-level air pollution and frailty among the elderly population in China. Health Place. 2020;64:102362. 32838887 10.1016/j.healthplace.2020.102362

[44] Kodavanti UP. Susceptibility variations in Air Pollution Health effects: incorporating neuroendocrine activation. Toxicol Pathol. 2019;47(8):962–75. 31594484 10.1177/0192623319878402 PMC9353182

[45] Jiang L, Chen X, Liang W, Zhang B. Alike but also different: a spatiotemporal analysis of the older populations in Zhejiang and Jilin provinces, China. BMC Public Health. 2023;23(1):1529. 37568136 10.1186/s12889-023-16433-w PMC10416386

[46] A look at. The lives of the elderly in the EU today is a web tool released by Eurostat, the statistical office of the European Union [https://ec.europa.eu/eurostat/cache/infographs/elderly/index.html

[47] Zhao S, Liu S, Hou X, Sun Y, Beazley R. Air pollution and cause-specific mortality: a comparative study of urban and rural areas in China. Chemosphere. 2021;262:127884. 33182102 10.1016/j.chemosphere.2020.127884

[48] Mueller N, Rojas-Rueda D, Basagaña X, Cirach M, Cole-Hunter T, Dadvand P, Donaire-Gonzalez D, Foraster M, Gascon M, Martinez D, et al. Urban and Transport Planning related exposures and mortality: a Health Impact Assessment for cities. Environ Health Perspect. 2017;125(1):89–96. 27346385 10.1289/EHP220 PMC5226698

[49] Glencross DA, Ho TR, Camiña N, Hawrylowicz CM, Pfeffer PE. Air pollution and its effects on the immune system. Free Radic Biol Med. 2020;151:56–68. 32007522 10.1016/j.freeradbiomed.2020.01.179

[50] Zhang L, Zeng X, He F, Huang X. Inflammatory biomarkers of frailty: a review. Exp Gerontol. 2023;179:112253. 37429425 10.1016/j.exger.2023.112253

[51] Karimi SM, Maziyaki A, Moghadam SA, Jafarkhani M, Zarei H, Moradi-Lakeh M, Pouran H. Continuous exposure to ambient air pollution and chronic diseases: prevalence, burden, and economic costs. Rev Environ Health. 2020;35(4):379–99. 32324166 10.1515/reveh-2019-0106

[52] Public Health and Aging. Trends in Aging—United States and Worldwide. JAMA. 2003;289(11):1371–3. 12636453

[53] Martens DS, Nawrot TS. Ageing at the level of telomeres in association to residential landscape and air pollution at home and work: a review of the current evidence. Toxicol Lett. 2018;298:42–52. 29944903 10.1016/j.toxlet.2018.06.1213

[54] Prüss-Üstün A, Wolf J, Corvalán C, Bos R, Neira M. Preventing disease through healthy environments: a global assessment of the burden of disease from environmental risks. World Health Organization, 2016.

[55] Beard JR, Bloom DE. Towards a comprehensive public health response to population ageing. Lancet. 2015;385(9968):658–61. 25468151 10.1016/S0140-6736(14)61461-6 PMC4663973

[56] Pedersen AB, Mikkelsen EM, Cronin-Fenton D, Kristensen NR, Pham TM, Pedersen L, Petersen I. Missing data and multiple imputation in clinical epidemiological research. Clin Epidemiol 2017:157–66. 10.2147/CLEP.S129785 PMC5358992 28352203

