# Supplementary material for: The association between PM2.5 and frailty: evidence from 122 cities in China and 7 countries in Europe
Source: BMC Public Health. 2024 Dec 30;24:3612. doi: 10.1186/s12889-024-21121-4 (PMC11684102; doi:10.1186/s12889-024-21121-4)
Supplement: Supplementary file 1 — Supplementary Material 1 [file 12889_2024_21121_MOESM1_ESM.docx]

**Supplemental Table**

| **Supplementary Table 1. List of health deficits items included in the frailty index** | | | |
| --- | --- | --- | --- |
| **No** | Description of the item | | Cut-off value |
|  | CHARLS | SHARE |  |
| **1** | Self-reported physician diagnosed hypertension | Self-reported physician diagnosed hypertension | Yes = 1, No = 0 |
| **2** | Self-reported physician diagnosed diabetes | Self-reported physician diagnosed diabetes | Yes = 1, No = 0 |
| **3** | Self-reported physician diagnosed cancer | Self-reported physician diagnosed cancer | Yes = 1, No = 0 |
| **4** | Self-reported physician diagnosed lung disease | Self-reported physician diagnosed lung disease | Yes = 1, No = 0 |
| **5** | Self-reported physician diagnosed arthritis | Self-reported physician diagnosed arthritis | Yes = 1, No = 0 |
| **6** | Self-reported physician diagnosed any emotional, nervous, or psychiatric problems | Self-reported physician diagnosed any emotional, nervous, or psychiatric problems | Yes = 1, No = 0 |
| **7** | Self-reported physician diagnosed memory-related disease | Self-reported physician diagnosed have Alzheimer's disease, dementia, organic brain syndrome, senility or any other serious memory impairment. | Yes = 1, No = 0 |
| **8** | Self-reported physician diagnosed heart problem | Self-reported physician diagnosed heart problem | Yes = 1, No = 0 |
| **9** | Self-reported physician diagnosed stroke | Self-reported physician diagnosed stroke | Yes = 1, No = 0 |
| **10** | Self-reported general health status | Self-reported general health status | CHARLS: Very poor or poor = 1; Very good, good, or fair =0; SHARE: Poor = 1; Fair, good, very good, excellent=0 |
| **11** | Difficulty with dressing | Difficulty with dressing | Yes = 1, No = 0 |
| **12** | Difficulty with bathing or showering | Difficulty with bathing or showering | Yes = 1, No = 0 |
| **13** | Difficulty with eating | Difficulty with eating | Yes = 1, No = 0 |
| **14** | Difficulty with getting in and out of bed | Difficulty with getting in and out of bed | Yes = 1, No = 0 |
| **15** | Difficulty with using the toilet | Difficulty with using the toilet | Yes = 1, No = 0 |
| **16** | Difficulty with managing money | Difficulty with managing money | Yes = 1, No = 0 |
| **17** | Difficulty with taking medication | Difficulty with taking medication | Yes = 1, No = 0 |
| **18** | Difficulty with shopping for groceries | Difficulty with shopping for groceries | Yes = 1, No = 0 |
| **19** | Difficulty with preparing meals | Difficulty with preparing meals | Yes = 1, No = 0 |
| **20** | Difficulty with cleaning house | Difficulty with doing work around the house and garden | Yes = 1, No = 0 |
| **21** | Mobility: difficulty with walking 100M | Mobility: difficulty with walking 100M | Yes = 1, No = 0 |
| **22** | Mobility: difficulty with getting up from a chair after sitting for long periods | Mobility: difficulty with getting up from a chair after sitting for long periods | Yes = 1, No = 0 |
| **23** | Mobility: difficulty with climbing several flights of stairs without resting | Mobility: difficulty with climbing several flights of stairs without resting | Yes = 1, No = 0 |
| **24** | Mobility: difficulty with lifting or carrying weights over 10 pounds/jins | Mobility: difficulty with lifting or carrying weights over 10 pounds/jins | Yes = 1, No = 0 |
| **25** | Mobility: difficulty with picking up a coin from the table | Mobility: difficulty with picking up a coin from the table | Yes = 1, No = 0 |
| **26** | Mobility: difficulty with stooping, kneeling, or crouching | Mobility: difficulty with stooping, kneeling, or crouching | Yes = 1, No = 0 |
| **27** | Mobility: difficulty with reaching arms above shoulder level | Mobility: difficulty with reaching arms above shoulder level | Yes = 1, No = 0 |
| **28** | Depression: CESD-10 questionnaire | Depression: EURO-D 12 questionnaire | Continuous variable, ranging from 0 to 1 |
| **29** | Cognition: Memory test score + Orientation test score + Serial 7's test score | | Continuous variable, ranging from 0 to 1 |

**Supplementary Table 2. The variables and the number of missing values that require multiple imputation***

| **Variables** | **Missing individuals, n (%)** | | | | | |
| --- | --- | --- | --- | --- | --- | --- |
|  | SHARE | | | CHARLS | | |
|  | Valid individuals | Missing individuals | Missing proportion | Valid individuals | Missing individuals | Missing proportion |
| **Marital status 2011** | 16915 | 114 | 0.67% | 6407 | 0 | 0.00% |
| **Marital status 2013** | 16916 | 113 | 0.66% | 6404 | 3 | 0.05% |
| **Marital status 2015** | 16923 | 106 | 0.62% | 6407 | 0 | 0.00% |
| **BMI 2011** | 16689 | 340 | 2.00% | 5696 | 711 | 11.10% |
| **BMI 2013** | 16764 | 265 | 1.56% | 5191 | 1216 | 18.98% |
| **BMI 2015** | 16800 | 229 | 1.34% | 5676 | 731 | 11.41% |
| **Drink status 2011** | 16969 | 60 | 0.35% | 6407 | 0 | 0.00% |
| **Drink status 2013** | 17021 | 8 | 0.05% | 6400 | 7 | 0.11% |
| **Drink status 2015** | 17028 | 1 | 0.01% | 6403 | 4 | 0.06% |
| **Smoke status 2011** | 16968 | 61 | 0.36% | 6406 | 1 | 0.02% |
| **Smoke status 2013** | 17028 | 1 | 0.01% | 4802 | 1605 | 25.05% |
| **Smoke status 2015** | 58 | 16971 | 99.66% | 6404 | 3 | 0.05% |
| **Retirement status 2011** | 16969 | 60 | 0.35% | 6339 | 68 | 1.06% |
| **Retirement status 2013** | 16928 | 101 | 0.59% | 6392 | 15 | 0.23% |
| **Retirement status 2015** | 16907 | 122 | 0.72% | 6398 | 9 | 0.14% |
| **Orient 2011** | 12207 | 4822 | 28.32% |  |  |  |
| **Orient 2013** | 65 | 16964 | 99.62% |  |  |  |
| **Orient 2015** | 16989 | 40 | 0.23% |  |  |  |

*The multiple imputation method to infer and fill in missing values using observed values from the existing data. By analyzing complete datasets and combining the results, the final outcomes were obtained. CHARLS: China Health and Retirement Longitudinal Study; SHARE: the Survey of Health, Aging, and Retirement in Europe.
